# External validation of the APPS, a new and simple outcome prediction score in patients with the acute respiratory distress syndrome

**DOI:** 10.1186/s13613-016-0190-0

**Published:** 2016-09-15

**Authors:** Lieuwe D. Bos, Laura R. Schouten, Olaf L. Cremer, David S. Y. Ong, Marcus J. Schultz, MARS consortium, Jos F. Frencken, Marc Bonten, Peter M. C. Klein Klouwenberg, David Ong, Roosmarijn T M. van Hooijdonk, Mischa A. Huson, Laura R A. Schouten, Marleen Straat, Lonneke A. van Vught, Maryse A. Wiewel, Esther Witteveen, Gerie J. Glas, Luuk Wieske, Tom van der Poll

**Affiliations:** 1 Department of Intensive Care, Academic Medical Center, Meibergdreef 9, 1105 AZ Amsterdam, The Netherlands; 2 Department of Intensive Care Medicine, University Medical Center Utrecht, Utrecht, The Netherlands; 3 Department of Medical Microbiology, University Medical Center Utrecht, Utrecht, The Netherlands

**Keywords:** ARDS, Prediction, Mortality, Sensitivity, Specificity, APPS

## Abstract

**Background:**

A recently developed prediction score based on age, arterial oxygen partial pressure to fractional inspired oxygen ratio (PaO_2_/FiO_2_) and plateau pressure (abbreviated as ‘APPS’) was shown to accurately predict mortality in patients diagnosed with the acute respiratory distress syndrome (ARDS). After thorough temporal external validation of the APPS, we tested the spatial external validity in a cohort of ARDS patients recruited during 3 years in two hospitals in the Netherlands.

**Methods:**

Consecutive patients with moderate or severe ARDS according to the Berlin definition were included in this observational multicenter cohort study from the mixed medical-surgical ICUs of two university hospitals. The APPS was calculated per patient with the maximal airway pressure instead of the plateau pressure as all patients were ventilated in pressure-controlled mode. The predictive accuracy for hospital mortality was evaluated by calculating the area under the receiver operating characteristics curve (AUC-ROC). Additionally, the score was recalibrated and reassessed.

**Results:**

In total, 439 patients with moderate or severe ARDS were analyzed. All-cause hospital mortality was 43 %. The APPS predicted all-cause hospital mortality with moderate accuracy, with an AUC-ROC of 0.62 [95 % confidence interval (CI) 0.56–0.67]. Calibration was moderate using the original cutoff values (Hosmer–Lemeshow goodness of fit *P* < 0.001), and recalibration was performed for the cutoff value for age and plateau pressure. This resulted in good calibration (*P* = 1.0), but predictive accuracy did not improve (AUC-ROC 0.63, 95 % CI 0.58–0.68).

**Conclusions:**

The predictive accuracy for all-cause hospital mortality of the APPS was moderate, also after recalibration of the score, and thus the APPS does not seem to be fitted for that purpose. The APPS might serve as simple tool for stratification of mortality in patients with moderate or severe ARDS. Without recalibrations, the performance of the APPS was moderate and we should therefore hesitate to blindly apply the score to other cohorts of ARDS patients.

**Electronic supplementary material:**

The online version of this article (doi:10.1186/s13613-016-0190-0) contains supplementary material, which is available to authorized users.

## Background

Outcome prediction in critically ill patients is commonly performed using general-purpose scoring systems such as the Acute Physiology and Chronic Health Evaluation (APACHE) score [1] and the Simplified Acute Physiology Score (SAPS) [2], which have been developed in unselected series of ICU patients. Other scoring systems have been developed for selective patient groups in the intensive care unit (ICU), e.g., for patients who develop acute kidney injury [3, 4] and liver failure [5].

Unfortunately, no such prediction system has been developed for patients with the acute respiratory distress syndrome (ARDS). Outcome prediction in patients with ARDS based on PaO_2_/FiO_2_, as proposed in the American-European Consensus Conference (AECC) criteria [6] and the Berlin definition for ARDS [7], does neither show good predictive accuracy nor show calibration [7–9]. Very recently, a scoring system was developed that predicts hospital mortality with good accuracy in patients with ARDS [10]. This score is based on three routinely available variables: age, the arterial oxygen partial pressure to fractional inspired oxygen ratio (PaO_2_/FiO_2_) and plateau pressure measured 24 h after the initial diagnosis of ARDS, and was thus coined the APPS. However, after excellent results of temporal external validation of this so-called APPS by the original authors, spatial external validation (e.g., the accuracy of prediction in another location) is highly needed.

Therefore, we tested the predictive accuracy and calibration of the APPS in a cohort of consecutive prospectively identified ARDS patients in two university hospitals in the Netherlands and recalibrated the score for our population of patients. We hypothesized that the ability of the APPS to predict hospital mortality remains excellent after spatial external validation.

## Methods

### Study design

The patient cohort was previously described by Geboers et al. [11]. Patients with ARDS, according to the Berlin definition, were selected from the parent ‘Molecular Diagnosis and Risk Stratification’ (MARS) study, performed in the ICUs of two tertiary care hospitals in the Netherlands (Academic Medical Center, Amsterdam, The Netherlands; University Medical Center, Utrecht, The Netherlands). The Medical Ethics Committees of both hospitals approved the study protocol and opt-out consent method. The patient or their legal representative was presented with a brochure and opt-out form, to be completed in case of unwillingness to participate.

### Setting

ICUs are closed-format units, with a team of board-certified critical care physicians, fellows in critical care medicine and board-certified ICU nurses caring for a mixed medical-surgical population of patients. The nurse-to-patient ratio was from 1:1 to 1:2. Patients received lung-protective mechanical ventilation per protocol, which mandated the use of low tidal volumes (6–8 mL/kg predicted body weight), a minimum level of positive end-expiratory pressure of 5 cmH_2_O, which together with FiO_2_ was titrated based on frequent PaO_2_ measurements. As part of standard care, nurses and attending physicians checked hourly whether there were signs of spontaneous breathing activity by comparing the set and measured respiratory rate and by observing flow curves at the ventilator. In case this was seen, the ventilator could be switched to an assisted ventilation mode, or additional sedation was given. Recruitment maneuvers and prone ventilation were used early and frequently if hypoxemia did not respond to higher levels of PEEP and FiO_2_. Details of the ventilation protocol were reported before [12]. A conservative fluid strategy was followed according to the ARDSnet protocol [13], and analgo-sedation was applied using sedation scales and bolus sedation with midazolam or continuous sedation with propofol. Details of the analgo-sedation protocol were also reported before [14]. Neuromuscular blocking agents were not routinely used, and if used only as a bolus.

### Inclusion and exclusion criteria

Consecutive adult patients admitted to the ICU with an expected length of stay of more than 24 h from January 2011 to December 2013 were eligible for participation in the MARS study. ARDS was defined according to the criteria stated by the American-European Consensus Conference on ARDS: i.e., the diagnosis required an acute onset of symptoms, the presence of bilateral infiltrates on chest radiography, a pulmonary-artery wedge pressure <18 mmHg and/or the absence of signs of left ventricular dysfunction, and a PaO_2_/FiO_2_ ≤ 200. Although our study started in 2011, before the recent ‘Berlin definition for ARDS’, we found that 100 % patients would have fulfilled the criteria of the new definition. Patients that were discharged or transferred to another ICU within 24 h after the diagnosis of ARDS were excluded from the present analysis, as they could not be used to validate the results reported by the ALIEN Network investigators. There were no additional inclusion or exclusion criteria for the present analysis. ARDS was diagnosed by a dedicated team of researchers who were trained in the proper use of the AECC criteria for ARDS [12]. The cause for ARDS was determined and scored in the following categories: pneumonia, aspiration, other pulmonary (i.e., inhalation trauma, near drowning), sepsis, trauma or major surgery, pancreatitis or other non-pulmonary (i.e., blood transfusion, toxic medication). In the event of multiple causes for ARDS, each cause was scored separately.

### APPS

The APPS was calculated as proposed in the original publication [10]. However, instead of plateau pressure, maximal airway pressure was used since pressure-controlled ventilation was used exclusively in our setting. The maximal airway pressure during pressure-controlled ventilation is equal to the plateau pressure during volume-controlled ventilation under most circumstances. As described above, nurses and physicians screened whether the ventilator could be switched to an assisted ventilation mode.

### Outcomes

All-cause in-hospital mortality was used as the primary endpoint. The data collectors were blind for this outcome at the moment of data collection as the all parameters were collected prospectively. If a patient was transferred to another hospital, that hospital was contacted to obtain the date of hospital discharge. Follow-up was complete for all patients.

### Statistical analysis

Data were expressed as mean ± SD, median with interquartile range or number with percentage, as appropriate. Differences between groups were tested with the Pearson Chi-square or Fisher exact test for categorical variables and with *T* test, one-way ANOVA, Mann–Whitney or Kruskal–Wallis test for numerical variables. A *P* value below 0.05 was considered significant. All analyses were performed in R via the R-studio interface.

The predictive performance of the APPS was assessed by quantifying the calibration and the accuracy of the score [15]. The predictive accuracy was expressed in the area under the receiver operating characteristics curve (AUC-ROC), and the predictive accuracy of the APPS was compared to the APACHE IV score. Sensitivity, specificity and likelihood ratios were calculated for the optimal cutoff obtained by the Youden index. A Kaplan–Meier curve was constructed for the APPS categories 3–4, 5–7, 8–9, as in the original report on the APPS [10]. Calibration was visualized by plotting the APPS against the percentage of non-survivors at that score and quantified by the Hosmer–Lemeshow goodness-of-fit test. Recalibration was performed manually, and measures of calibration and predictive accuracy were reassessed. A sensitivity analysis was performed in patients that received mechanical ventilation according to the ventilation protocol in the derivation study for the APPS (i.e., patients were ventilated using the following settings: PEEP ≥ 10 cmH_2_O and FiO_2_ ≥ 50 %). A *P* value below 0.05 was considered significant. All analyses were performed in R via the R-studio interface.

## Results

**Fig. 1:**
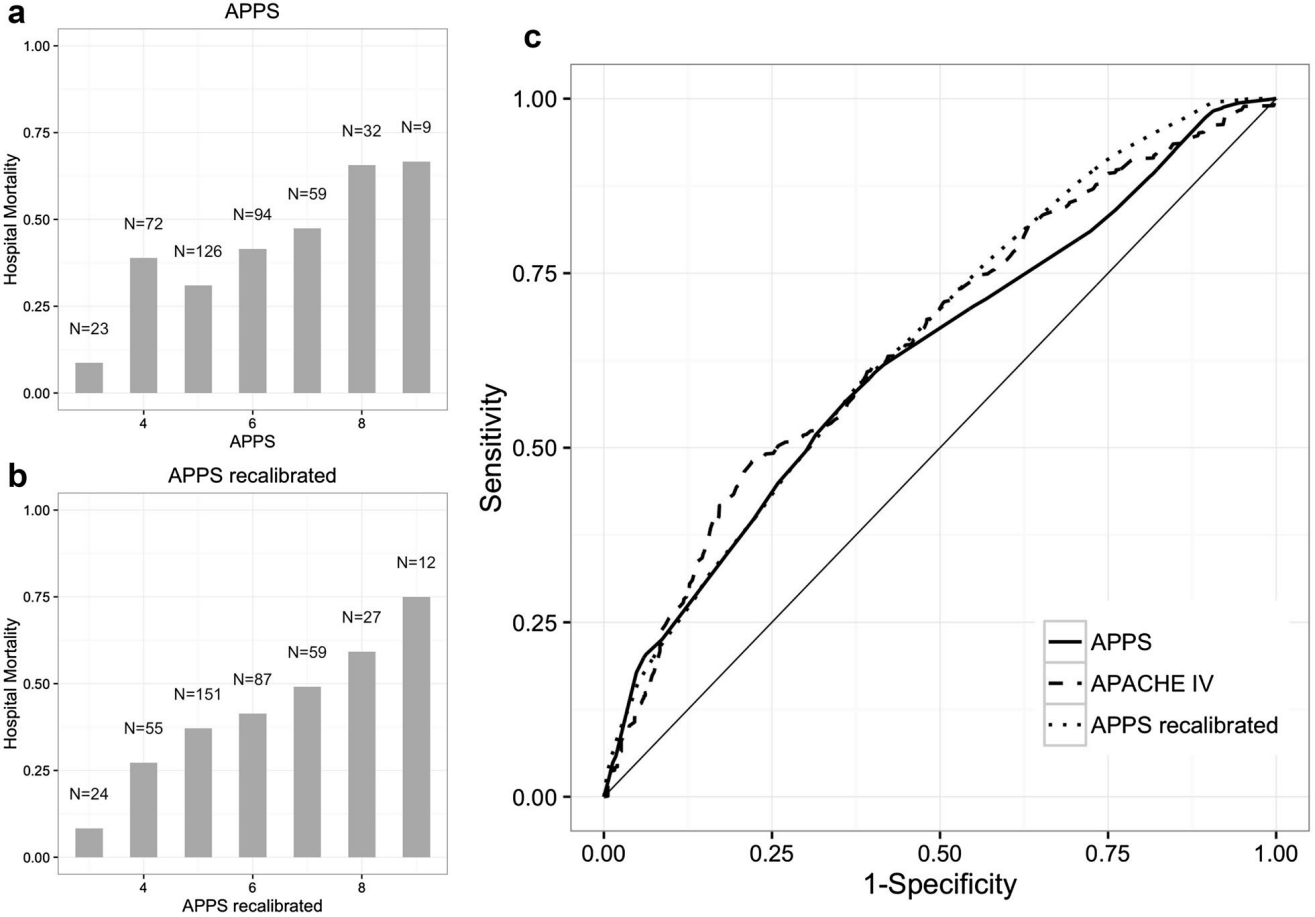
Calibration and predictive accuracy of the APPS for hospital mortality. **a** Original APPS. **b** Recalibrated APPS. **c** ROC curves. Each *bar* represents the percentage of patients that did not survive that hospital admission. The *number* indicates the total number of patients with that APPS

The cohort consisted of 439 patients with moderate or severe ARDS. Baseline characteristics are described in Table 1. Pressure-controlled ventilation was exclusively used; indeed, volume-controlled ventilation and assisted ventilation modes were not used at the moments data were collected for the present investigation. All-cause hospital mortality was 43 %. The mean APPS was 5 in surviving patients and 6 in non-surviving patients (Additional file 1: Figure E1; *P* < 0.001). The APPS predicted all-cause hospital mortality with moderate accuracy with an AUC-ROC of 0.62 (95 % confidence interval 0.56–0.67, see Fig. 1; Table 2), which was not significantly different from the predictive value of the APACHE IV score (AUC-ROC 0.66, 95 % CI 0.61–0.71; *P* = 0.22). The APPS showed a disturbed calibration at a score of 4–5 (Fig. 1; *P* < 0.001). This was mainly due to the categorization of the variables age and Pmax (Table 3, Additional file 1: Figure E2). This was translated into overlapping Kaplan–Meier curves for the APPS categories 3–4 and 5–7 (Additional file 1: Figure E3).

**Table 1:** Baseline characteristics of 439 survivors and non-survivors with the acute respiratory distress syndrome in the Netherlands

	Survivors (*N* = 252)	Non-survivors (*N* = 187; 43 %)	*P*
Gender, male, *N* (%)	163 (64.7)	120 (64.2)	0.92
Age, mean ± SD	58.5 ± 15.4	63.1 ± 12.7	0.001
Cause of ARDS, *N* (%)			
Pneumonia	154 (61.1)	115 (61.5)	1.0
Aspiration	25 (9.9)	16 (8.6)	0.76
Other pulmonary	2 (0.8)	1 (0.5)	1.0
Sepsis	144 (57.1)	132 (70.6)	0.003
Trauma	38 (15.1)	15 (8.0)	0.029
Pancreatitis	2 (0.8)	6 (3.2)	0.069
Other non-pulmonary	29 (11.5)	17 (9.1)	0.43
Disease severity, mean ± SD			
APACHE IV	85.5 ± 27	102.7 ± 30.7	<0.001
SOFA score	8.6 ± 3.2	10.1 ± 4.1	<0.001
Physiological parameters, mean ± SD			
pH, median ± IQR	7.4, 7.4–7.5	7.4, 7.3–7.4	0.001
PaCO_2_	42.1 ± 9	44.4 ± 12.1	0.039
PaO_2_/FiO_2_	126.8 ± 38.3	127.7 ± 43.1	0.81
Respiratory system compliance	28.9 ± 15.6	37.4 ± 20.9	<0.001
Ventilation parameters, mean ± SD			
Tidal volume (ml/kg PBW)	7.7 ± 2	7.5 ± 1.7	0.38
FiO_2_	53.2 ± 12.9	56.7 ± 16.7	0.017
Respiratory rate	22 ± 7	25 ± 8	<0.001
PEEP (cmH_2_O)	10.4 ± 3.6	10.9 ± 4	0.2
*P*_max_ (cmH_2_O)	26.2 ± 7.9	28.2 ± 9.4	0.018

**Table 2:** Test characteristics

	ROC	CI	Cutoff	Sens	Spec	LR+	LR−
Complete cohort (*N* = 439)							
APPS	0.62	0.56–0.67	5.5	0.63	0.56	1.43	0.66
Recalibrated APPS	0.63	0.58–0.68	5.5	0.63	0.56	1.43	0.66
Sensitivity analysis (*N* = 151)							
APPS	0.62	0.54–0.71	5.5	0.38	0.85	2.53	0.73
Recalibrated APPS	0.64	0.55–0.73	5.5	0.48	0.78	2.18	0.37

*ROC* receiver operating characteristics curve, *CI* 95 % confidence interval of area under the ROC curve, *Sens* sensitivity, *Spec* specificity, *LR* likelihood ratio

**Table 3:** Odds ratios per category APPS

Variable	Range	Category	*N*	Hospital mortality (%)	OR	OR 2.5 %	OR 97.5 %	*P* for trend
Age	<47	1	72	26.4	1			0.0046
	47–66	2	196	43.9	2.18	1.2	3.95	
	>66	3	171	48	2.57	1.41	4.7	
PaO_2_/FiO_2_	>158	1	239	36.4	1			0.0015
	105–158	2	135	46.7	1.53	1	2.35	
	<105	3	65	56.9	2.31	1.32	4.03	
*P*_max_	<27	1	233	34.8	1			0.0021
	27–30	2	48	25	0.63	0.31	1.27	
	>33	3	134	52.2	2.05	1.33	3.17	

Recalibration was performed for two of the three facets of the APPS. The age limit for 2 points was set to 47 and for 3 points to above 59 years (see Table 4). A maximum airway pressure above 30 resulted in 2 points and above 33 in 3 points. This resulted in good calibration (Fig. 1; Table 4; Additional file 1: Figure E4, E5, *P* = 1.0), but predictive accuracy remained moderate (AUC-ROC 0.63, 95 % CI 0.58–0.68, Fig. 1). Survival was significantly different when the APPS categories were changed to 3, 4–7 and 8–9 (*P* < 0.001, Additional file 1: Figure E6).

**Table 4:** Odds ratios per category recalibrated APPS

Variable	Range	Category	*N*	Hospital mortality (%)	OR	OR 2.5 %	OR 97.5 %	*P* for trend
Age	<47	1	72	26.4	1			0.0021
	47–59	2	96	41.7	1.99	1.03	3.87	
	>59	3	271	47.2	2.5	1.4	4.44	
PaO_2_/FiO_2_	>158	1	239	36.4	1			0.0015
	105–158	2	135	46.7	1.53	1.00	2.35	
	<105	3	65	56.9	2.31	1.32	4.03	
*P*_max_	<30	1	281	33.1	1			0.0001
	30–33	2	40	45	1.65	0.85	3.23	
	>33	3	94	55.3	2.5	1.55	4.03	

A sensitivity analysis was limited to patients that were ventilated following the protocol that was used in the derivation cohort (*N* = 151), where the ventilation data were collected under the following standardized ventilatory settings: PEEP ≥ 10 cmH_2_O and FiO_2_ ≥ 50 %. This analysis confirmed a moderate predictive accuracy for the original (AUC-ROC 0.62, 95 % CI 0.54–0.71) and the recalibrated APPS (AUC-ROC 0.64, 95 % CI 0.55–0.73).

## Discussion

Spatial external validation of the APPS in two university hospitals in the Netherlands showed a considerable lower predictive accuracy for all-cause hospital mortality than in the derivation and temporal validation population in the Spanish hospitals. Calibration was also disturbed, but this was resolved after minor modification of the score.

Patient characteristics were strikingly similar in both studies. For example, hospital mortality was comparable between the cohorts (46 % in the derivation cohort, 42 % in temporal validation cohort and 43 % in spatial validation cohort). Furthermore, ventilator parameters were also comparable, with the exception of FiO_2_ (80 % in derivation and temporal validation cohorts, 60 % in spatial validation cohort). Additionally, the strength of the association between aspects of the APPS and mortality, as exemplified by the odds ratio (Tables 2, 3), was similar between the cohorts. Importantly, the odds ratio is a measure of effect size and not of discrimination. This implies that the association between hospital mortality and age, PaO_2_/FiO_2_ and plateau pressure was very similar between the cohorts, but that this did not result in sufficient discrimination in the population we included.

Any difference in patient selection, practice or data collection between the temporal validation and spatial validation cohorts may explain the differences in discrimination. First, it could be argued that differences arose because we used the maximal airway pressure instead of the plateau pressure. Although the maximal airway pressure can be used to approximate the plateau pressure in theory [16], it could be that, for example, during undetected spontaneous breathing effort these values were influenced [17]. In our setting, however, nurses and physicians carefully and hourly check whether a patient is breathing spontaneously. If so, the local ventilation protocol dictates the use of an assisted ventilation mode, and this was not seen at the moments of data collection for this study. The maximal airway pressure and the plateau pressure are both surrogate measures for alveolar distending pressure, and the accuracy of the score may improve if that pressure would be measured directly. PaO_2_/FiO_2_ may be influenced by ventilator settings [8], and therefore we performed a sensitivity analyses for patients that were using the standardized ventilator settings (PEEP ≥ 10 cmH_2_O and FiO_2_ ≥ 50 %) that were used in the original study. However, this did not change the results. This implies that differences in ventilation strategies are not likely to have caused the lower predictive accuracy. Thus, the APPS may have been over-fitted to the setting in which it is developed and validation. This observation is further supported by the observation that not only maximal airway pressure and PaO_2_/FiO_2_ discriminated differently between the cohorts, but that this lower accuracy was also found for age. In contrast to the former, data collection will not influence the age of the patient. Thereby, we can establish that the lower accuracy may partly be due to differences in data collection, but also that the APPS cannot be generalized to other populations due to over-fitting to the derivation population.

The presented data suggest that calibration of the APPS is sufficiently good after slight modification of the original score. Calibration may be more important than predictive accuracy for some purposes. For example, for inclusion into clinical trials the added value of discrimination is limited, while calibration is pivotal. A well-calibrated score could lead to the inclusion of a patient population with the mortality to which the study is powered (prognostic enrichment), something that has been an issue in many investigational trials [18–20]. However, it is worrisome that recalibration of the cutoffs for age and pressure was needed as this limits the implementation of the score in new clinical environments. Additional validation attempts could further clarify the optimal cutoffs for the score and may allow for stratification of newly recruited ARDS patients.

Based on our data, the validity of the APPS as a prediction score for mortality in ARDS is disputable. But what purpose would a prediction score for mortality serve? The authors that proposed the APPS suggest that the score may be used to identify patients in whom benefit from the treatment may be limited. However, here the same point can be made as in the previous paragraph; it may be sufficient to identify groups of patients that have a higher or lower mortality and treat those groups differently. A well-calibrated score will serve this point, and for that purpose, the APPS may still qualify. It could be argued that we should have improved the prediction score. However, this was not the aim of this study. Thorough validation of well-developed scores is more important than development of multiple prediction tools [21]. The two-center, single national design is another limitation of the present study as ideally the accuracy of a predictive test such as the APPS is validated in a prospective, international observational cohort study.

To conclude, our data suggest the APPS could serve as simple tool for stratification of mortality in patients with moderate or severe ARDS. Importantly, without recalibrations the performance of the APPS was moderate and we should therefore hesitate to blindly apply the score to new series of patients. The predictive accuracy for all-cause hospital mortality was moderate, also after recalibration of the score, and thus the APPS does not seem to be fitted for that purpose.

## Appendix: Members of the MARS consortium

Jos F. Frencken (Department of Intensive Care Medicine and Julius Center for Health Sciences and Primary Care, University Medical Center Utrecht, Utrecht, the Netherlands); Marc Bonten, Peter M. C. Klein Klouwenberg, David Ong (Department of Medical Microbiology, University Medical Center Utrecht, Utrecht, the Netherlands); and Roosmarijn T. M. van Hooijdonk, Mischa A. Huson, Laura R. A. Schouten, Marleen Straat, Lonneke A. van Vught, Maryse A. Wiewel, Esther Witteveen, Gerie J. Glas, and Luuk Wieske (Department of Intensive Care Medicine, Academic Medical Center, University of Amsterdam); Tom van der Poll (Center of Experimental Molecular Medicine; CEMM, Academic Medical Center, University of Amsterdam).

## Additional file

10.1186/s13613-016-0190-0 Additional methods and results.
