# Supplementary material for: External validation of the APPS, a new and simple outcome prediction score in patients with the acute respiratory distress syndrome
Source: Ann Intensive Care. 2016 Sep 15;6:89. doi: 10.1186/s13613-016-0190-0 (PMC5023650; doi:10.1186/s13613-016-0190-0)

**Online Supplement**

**Supplemental tables:**

**Table E1: Logistic regression model with all physiological variables.**

|  | Coefficients | P |
| --- | --- | --- |
| Intercept | -3.204 | 0.0004 |
| Age | 0.029 | 0.0081 |
| PaO2/FiO2 | -0.005 | 0.0002 |
| Pmax | 0.071 | 0.0024 |
| PEEP | -0.135 | 0.6426 |
| TV/IBW | 0.028 | <0.001 |
| SOFA | 0.128 | <0.001 |

**Supplemental figures**

Figure E1: Distribution of APPS and attributes for survivors and non-survivors of that hospital admission.

**
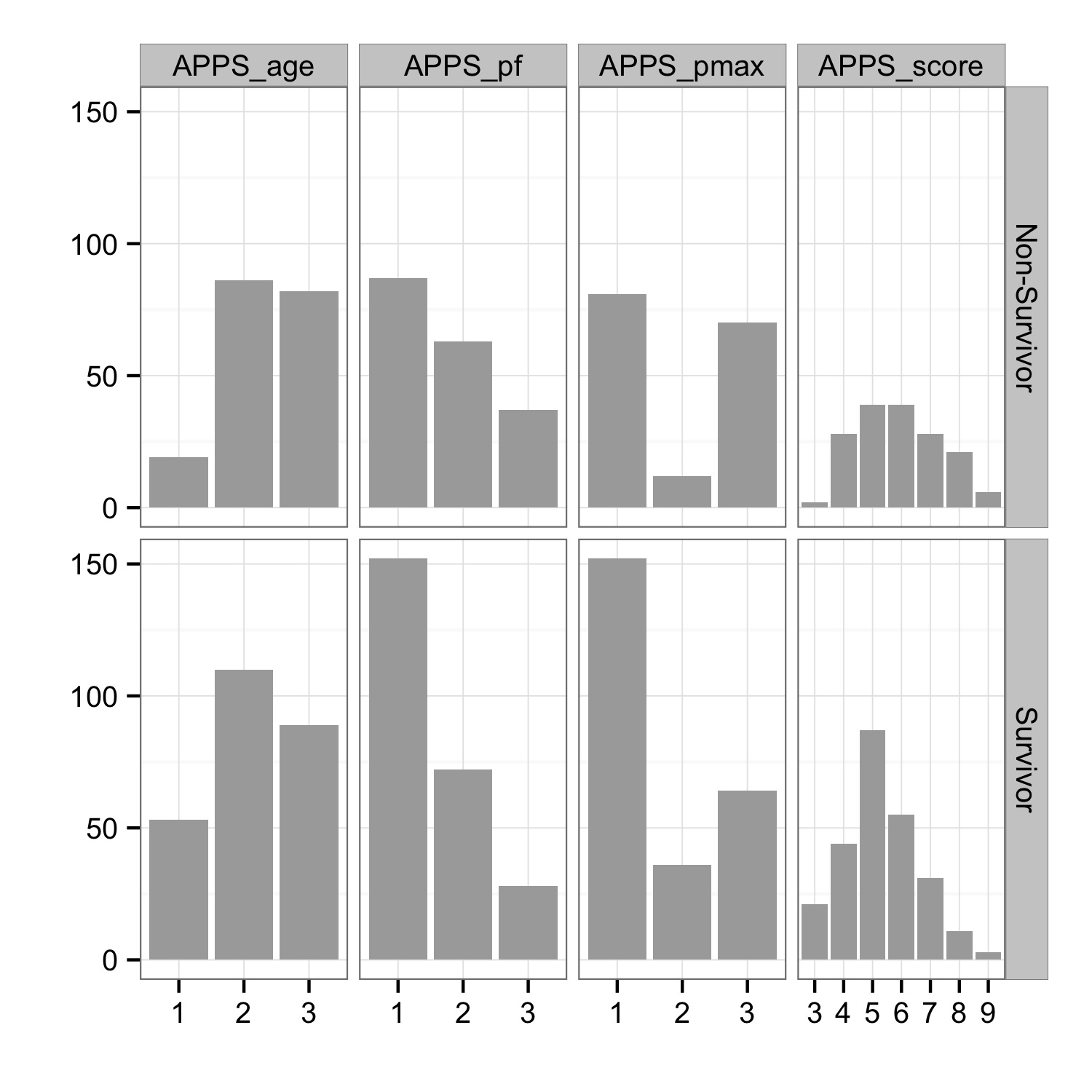
**

Figure E2: Occurrence of hospital mortality per APPS and attributes.


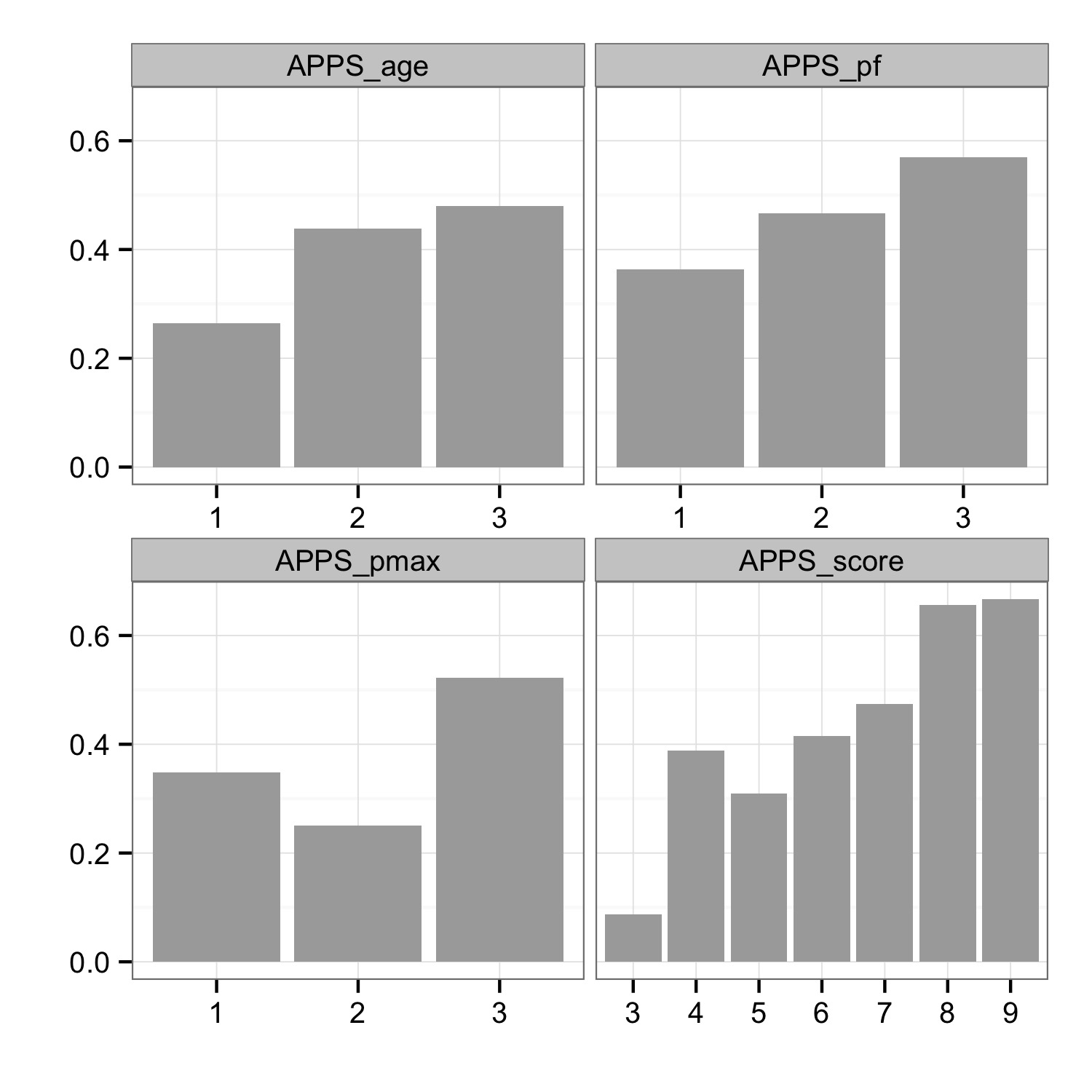


Figure E3: Kaplan-Meijer curve per APPS category.


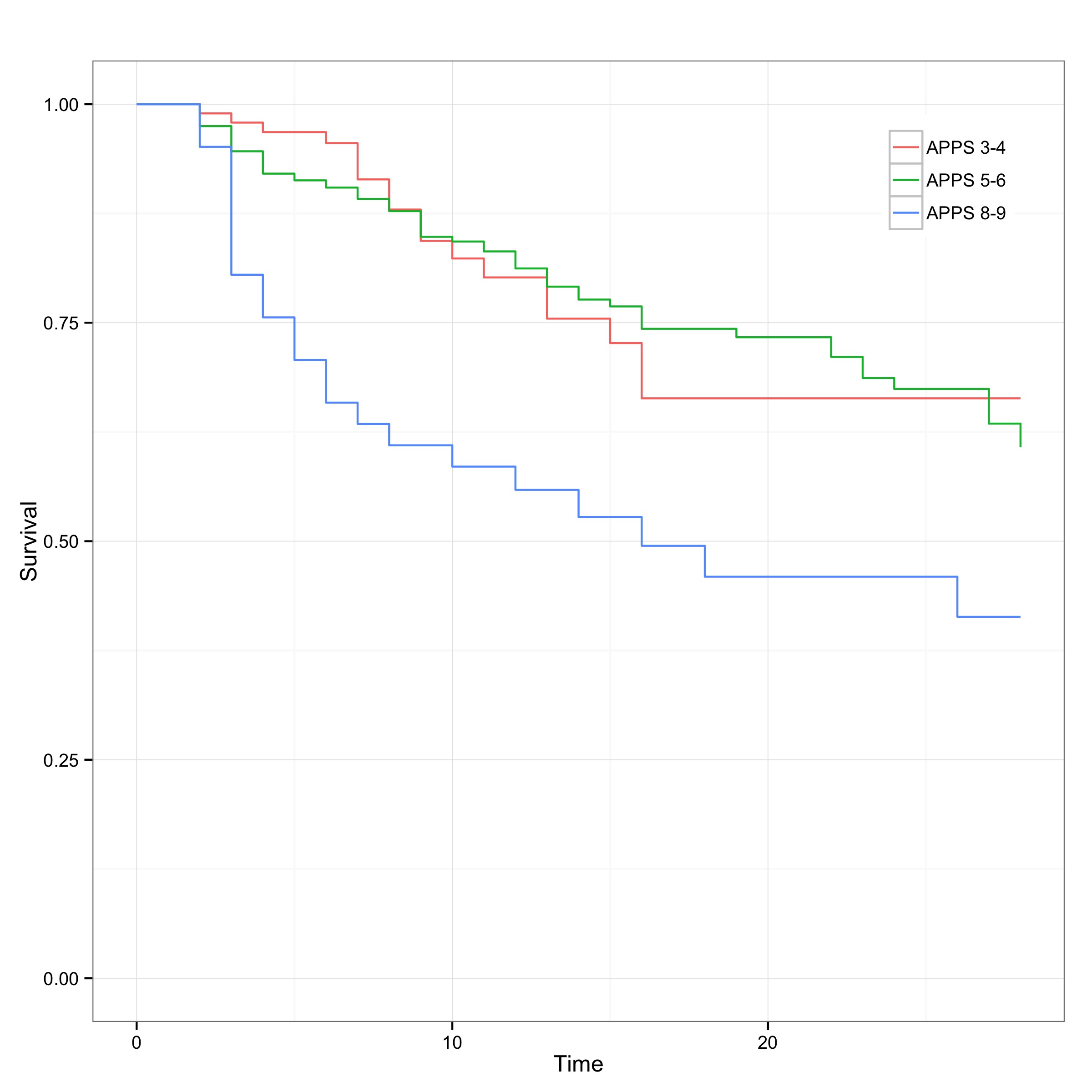


Figure E4: Distribution of APPS and attributes after recalibration for survivors and non-survivors of that hospital admission.


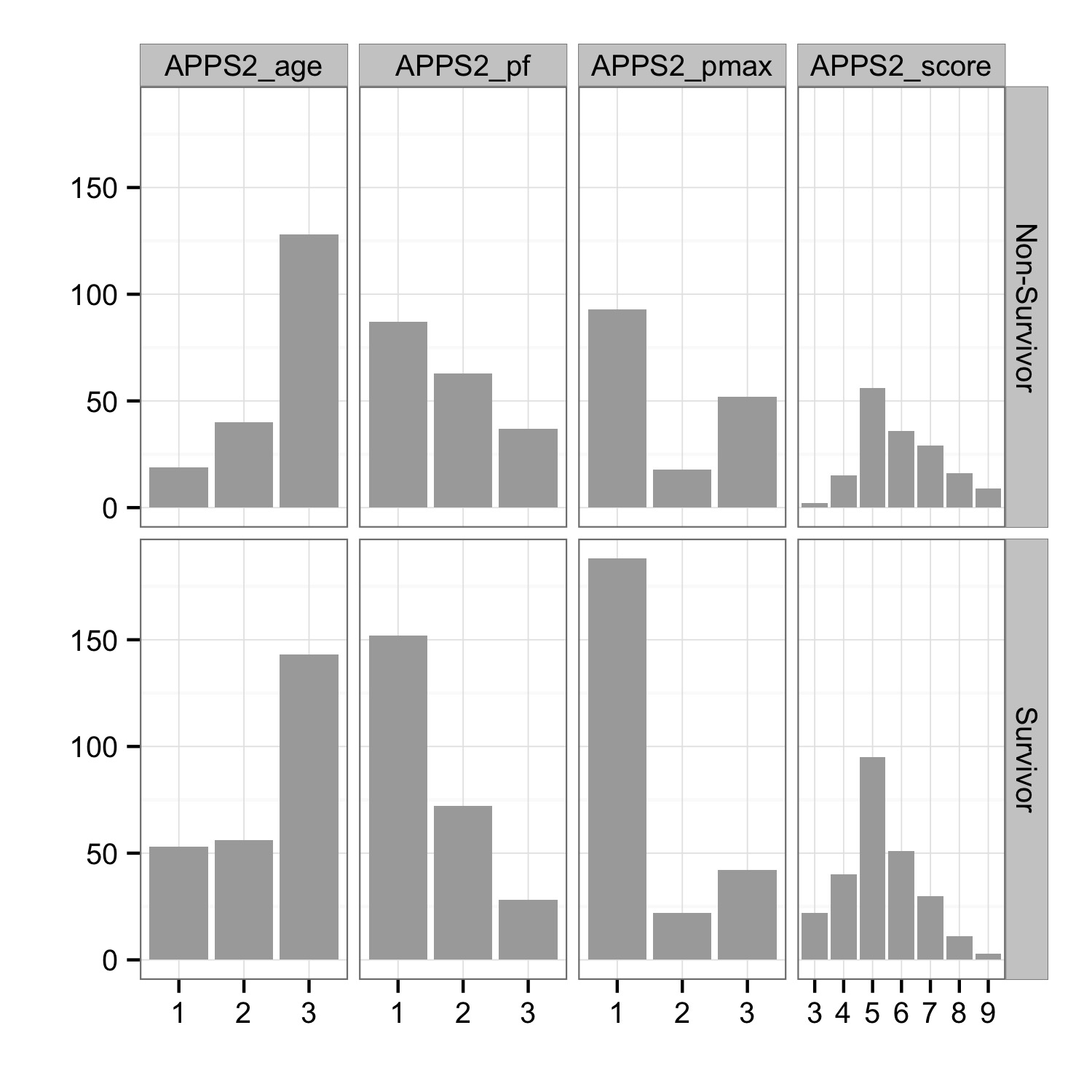


Figure E5: Occurrence of hospital mortality per APPS and attributes after recalibration.

**
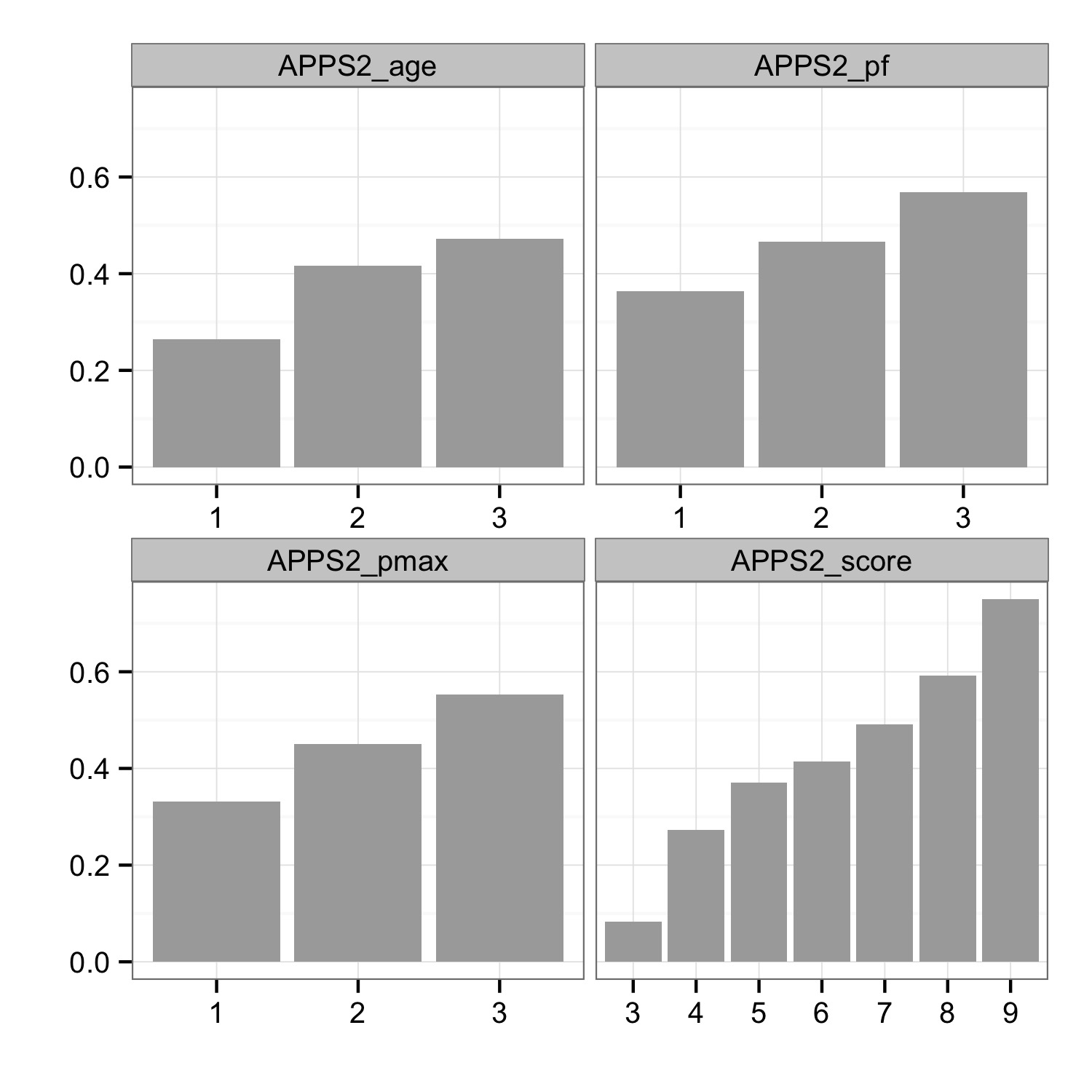
**

Figure E6: Kaplan-Meijer curve per recalibrated APPS category.


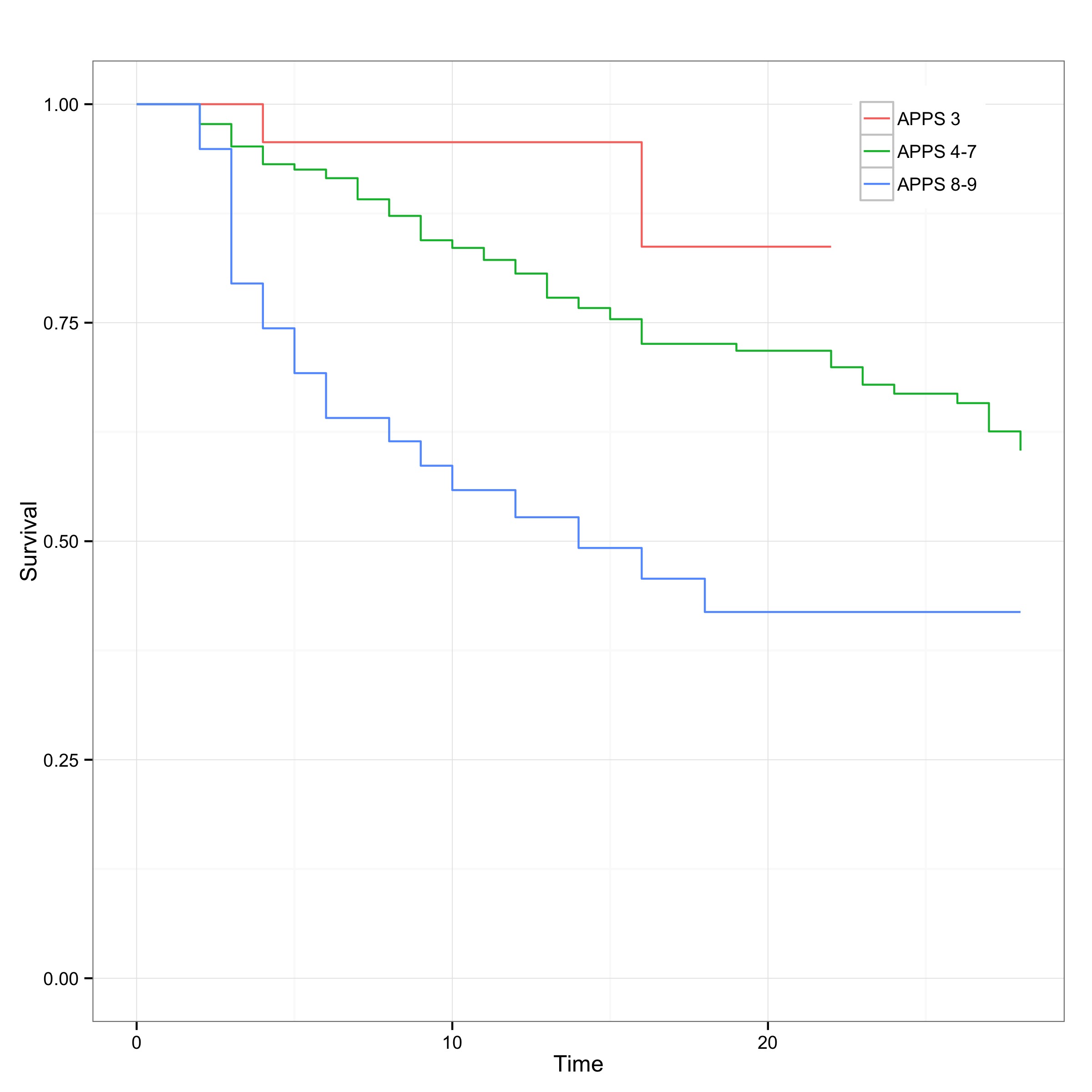

Supplement: Supplementary file 1 — 10.1186/s13613-016-0190-0 Additional methods and results. [file 13613_2016_190_MOESM1_ESM.docx]
